# Promises and pitfalls of a Pannexin1 transgenic mouse line

**DOI:** 10.3389/fphar.2013.00061

**Published:** 2013-05-09

**Authors:** Regina Hanstein, Hiromitsu Negoro, Naman K. Patel, Anne Charollais, Paolo Meda, David C. Spray, Sylvia O. Suadicani, Eliana Scemes

**Affiliations:** ^1^Dominick P. Purpura Department of Neuroscience, Albert Einstein College of Medicine, Yeshiva UniversityNew York, NY, USA; ^2^Department of Urology, Albert Einstein College of Medicine, Yeshiva UniversityNew York, NY, USA; ^3^Department of Cell Physiology and Metabolism, Medical School, University of GenevaGeneva, Switzerland

**Keywords:** qPCR, cell-specific deletion, Cre-recombinase, hypomorphism, pannexin

## Abstract

Gene targeting strategies have become a powerful technology for elucidating mammalian gene function. The recently generated knockout (KO)-first strategy produces a KO at the RNA processing level and also allows for the generation of conditional KO alleles by combining FLP/FRT and Cre/loxP systems, thereby providing high flexibility in gene manipulation. However, this multipurpose KO-first cassette might produce hypomorphic rather than complete KOs if the RNA processing module is bypassed. Moreover, the generation of a conditional phenotype is also dependent on specific activity of Cre recombinase. Here, we report the use of an efficient molecular biological approach to test pannexin1 (*Panx1*) mRNA expression in global and conditional Panx1 KO mice derived from the KO-first mouse line, Panx1^tm1a(KOMP)Wtsi^. Using qRT-PCR, we demonstrate that tissues from wild-type (WT) mice show a range of *Panx1* mRNA expression levels, with highest expression in trigeminal ganglia, bladder and spleen. Unexpectedly, we found that in mice homozygous for the KO-first allele, *Panx1* mRNA expression is not abolished but reduced by 70% compared to that of WT tissues. Thus, Panx1 KO-first mice present a hypomorphic phenotype. Crosses of Panx1 KO-first with FLP deleter mice generated Panx1^f/f^ mice. Further crosses of the latter mice with mGFAP-Cre or NFH-Cre mice were used to generate astrocyte- and neuron-specific Panx1 deletions, respectively. A high incidence of ectopic Cre expression was found in offspring of both types of conditional Panx1 KO mice. Our study demonstrates that *Panx1* expression levels in the global and conditional Panx1 KO mice derived from KO-first mouse lines must be carefully characterized to ensure modulation of *Panx1* gene expression. The precise quantitation of *Panx1* expression and its relation to function is expected to provide a foundation for future efforts aimed at deciphering the role of Panx1 under physiological and pathological conditions.

## Introduction

Pannexins are a group of proteins that share some sequence homology with the invertebrate gap junctions, the innexins, and because of that are considered to be members of this family. Three Pannexins (Panx1, Panx2, and Panx3) are present in mammalian tissues (Panchin et al., 2000). They have no sequence homologies with the chordate gap junction proteins, connexins, but membrane topology predicts similar four transmembrane domains with cytosolic N- and C- termini. Panx1 is ubiquitously expressed while Panx2 is restricted to the CNS and Panx3 is mainly found in cartilage and dermis (Baranova et al., 2004; Barbe et al., 2006; Penuela et al., 2007). Of the three, Panx1 is best characterized, forming high conductance plasma membrane channels with a maximal conductance of 500 pS that are permeable to ATP and modulated by intracellular signaling molecules (calcium, tyrosine kinase, caspases) (Bao et al., 2004; Locovei et al., 2006a; Pelegrin and Surprenant, 2006; Iglesias et al., 2008; Sandilos and Bayliss, 2012). Panx1 channel activity can be modulated by mechanical stretch, membrane potential, cytoplasmic Ca^2+^ concentration (Bao et al., 2004; Locovei et al., 2006b), and by ATP directly or via purinergic receptors (Locovei et al., 2006b, 2007; Pelegrin and Surprenant, 2006; Qiu and Dahl, 2009). Panx1 channels are permeable to ATP (Bao et al., 2004; Locovei et al., 2006a) and thus contribute to purinergic signaling including that involved in the propagation of intercellular calcium waves between astrocytes (Scemes et al., 2007; Suadicani et al., 2012), communication between taste bud cells (Huang et al., 2007), neutrophil activation and immune defense (Chen et al., 2010), and vascular tone (Billaud et al., 2012). Several studies indicate the involvement of Panx1 in certain pathophysiological conditions (ischemia, seizures, innate immune response, ATP-induced cell death, HIV infection, etc.) [reviewed in Scemes et al., 2009; Dahl and Keane, 2012].

Over the last few years at least four different transgenic mouse lines have been generated to knockdown Panx1 expression: the Monyer (single and double Panx1 and Panx2 KO (Bargiotas et al., 2011), the Knockout Mouse Project (KOMP; www.KOMP.org), the Genentech (www.genentech.com), and the Miami (Romanov et al., 2012) mice. While the former mice were generated by introducing a lacZ and a neomycin cassette within exon 1 of *Panx1* (Bargiotas et al., 2011), hence disrupting the gene transcription, the other three Panx1-null mice were designed using approaches that allow for both global deletion as well as cell-specific deletion. The Genentech and the Miami mouse lines were generated using the “conditional first” strategy, which relies on the creation of a conditional allele which when crossed with a Cre-deleter or a promoter specific-Cre mouse removes the loxP flanked region for transmission of the knockout (KO) or conditional allele. The KOMP mouse is based on the KO-first strategy (Testa et al., 2004) involving the insertion of a cassette into the first intron of *Panx1* that produces a KO at the transcript level, due to the presence of a splice acceptor in the cassette that captures the transcript. Thus, mice homozygous for the KO-first cassette are Panx1-null; however, a hypomorphic phenotype may result if the KO function of the RNA processing module is bypassed. For cell-specific KO, the KO-first allele was designed with two FRT sites flanking the cassette containing the splice acceptor and loxP sites flanking *Panx1* exon 2. Conditional KO mice can be generated by crossing Panx1 KO-first mice with flippase deleter mice, hereby inducing excision of the cassette at the FRT sites. This restores gene function and leaves *Panx1* exon 2 flanked by 2 loxP sites. The use of appropriate Cre expressing mouse lines then allows for a cell-type specific *Panx1* gene deletion.

Here we provide a molecular biological approach that allows for the evaluation of the state of knockdown of *Panx1* in the global and conditional Panx1 KO mice from KOMP. Our results indicate that global Panx1 KO mice (homozygous KO-first alleles) have a hypomorphic phenotype, with about 70% reduction of *Panx1* mRNA in 10 tissues that were analyzed. In the conditional Panx1 KO, our study indicates significant ectopic expression of Cre recombinase when using either mGFAP or the mNFH promoters to generate glia- and neuron-specific deletion of *Panx1*, respectively. We also describe a useful tail qRT-PCR method to readily detect such ectopic activity.

## Materials and methods

### Animals

The Panx1-null mouse line (Panx1^tm1a(KOMP)Wtsi^), generated by KOMP (www.KOMP.org) in the C57BL/6 background, uses a construct that introduces a floxed locus so that cell-type specific KO can be achieved through breeding with a Cre recombinase mouse. KOMP mice are maintained in our animal facility at Albert Einstein College of Medicine as global Panx1 knockout (Panx1 KO-first) and wild-type (WT) Panx1 (Panx1^+/+^). Panx1^f/f^ mice were generated by crossing Panx1 KO-first with flippase deleter mice (B6.ACTFLPe/J) to allow targeted knockdown of Panx1 in either astrocytes or neurons after crossing with cell-type specific Cre mice. For that, mGFAP-Cre (B6.Cg-Tg(Gfap-cre)73.12Mvs/J) and mNFH-Cre (Tg(Nefh-cre)12Kul/J) mice in the C57BL/6 background were purchased from Jackson laboratory and were used to generate mGFAP-Cre:Panx1^f/f^ and mNFH-Cre:Panx1^f/f^ mice that are maintained in our animal facility. For some studies, we also used the Panx1^−/−^ mouse line (Bargiotas et al., 2011), which was maintained at the animal facility of the University of Geneva. All studies were performed following protocols approved by the Albert Einstein Animal Care and Use Committee.

### Genotyping

**Figure 1:**
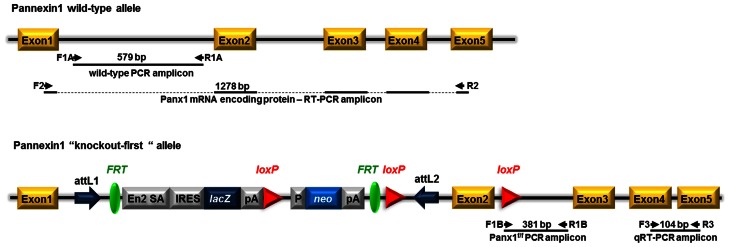
**Schematic view of the Panx1 wild-type allele and the “knockout-first” conditional allele of Panx1 ^tm1a(KOMP)Wtsi^ mice.** Pannexin1 (*Panx1*) gene consists of 5 exons. Panx1 “knockout-first” allele was generated by KOMP through insertion of the L1L2_Bact_P cassette into the mouse *Panx1* gene at position 15010456 of Chromosome 9. The cassette is composed of 2 FRT sites flanking an IRES:*lacZ* trapping cassette and a floxed human beta actin promoter-driven *neo* cassette inserted into the intron 1 of *Panx1* and an additional third loxP site downstream, at position 15009768, of *Panx1* exon 2, the critical exon. This “knockout-first” allele is designed to generate a Panx1 null allele through splicing exon 1 to a *lacZ* trapping element and disrupting *Panx1* mRNA expression. The trapping cassette also includes the mouse En2 splice acceptor (En2 SA) and the SV40 polyadenylation sequences. Position of primers used for genotyping (two primer sets: F1a—R1a and F1b—R1b), for RT-PCR (primer set F2—R2), and for qRT-PCR (primer set F3—R3) with expected amplicon length are indicated. *attL1* and *attL2*: sites for site-specific recombination of the entry clone. FRT: sites for flippase activity. EN2-SA: splice acceptor of mouse engrail (En2) exon 2. *IRES, internal ribosome entry site;* lacZ, gene encoding β-galactosidase; pA, polyadenylation signal; neo, neomycin phosphotransferase; loxP sites (triangles); target sites for Cre recombinase. Adapted from www.KOMP.org.

As previously described (Santiago et al., 2011), Panx1 KO-first mice were genotyped by tail PCR using two forward (F1a, F1b) and two reverse (R1a, R1b) primers (F1a: GAGATGGCGCAACGCAATTAAT; R1a: CTGGCTCTCATAATTCTTGCCCTG; F1b: CTGTATCACACAACCACTTCAGAGAAGG; R1b: GAGCTGACCCCTTTCCATTCAATAG3). The WT *Panx1* allele was targeted by primers F1a and R1a and identified as a 579 bp amplicon, while the transgene was targeted by primers F1b and R1b and identified as a 381 bp amplicon (Figure 1).

### Tissue preparation

Six month old WT and Panx1 KO-first mice were anesthetized with isoflurane and exsanguinated by intracardiac perfusion with ice-cold phosphate buffered saline, pH 7.4. The tissues were immediately removed, transferred to vials with RNA*later* solution (Ambion, Life Technologies, Grand Island, NY), and stored at 4°C until processing for qRT-PCR analysis. Tissue samples were collected from tail tips, nervous system (cortex, hippocampus, cerebellum, trigeminal ganglia), heart (apex region), bone (calvaria), spleen, urinary bladder, liver (middle lobe), and kidney (cortical and medullar regions).

### RT-PCR

Primers used for *Panx1* mRNA coding region were F: ATGGCCATCGCCCACTTG R: GCAGGACGGATTCAGAAGCC (1278 bp). Reaction mixtures using Multiplex PCR kit (Qiagen) with targeted cDNA were denatured at 95°C for 10 min, followed by 40 PCR cycles. Each cycle consisted of the following three steps: 94°C for 30 s, 55°C for 30 s, and 72°C for 90 s. Final extension was set at 72°C for 10 min.

### qRT-PCR

Tissues of adult mice (Panx1 WT, Panx1 KO-first, Panx^f/f^, GFAP-Cre:Panx1^f/f^, and NFH-Cre:Panx^f/f^) were used to quantify the levels of *Panx1* transcripts. Tissues were minced and homogenized with a Bullet Blender (Next Advance Inc.) and total RNA was extracted using the RNeasy fibrous tissue or plus mini kits (Qiagen) according to the manufacturer's protocol. Complementary DNA was synthesized from 1 μg/10 μl of RNA, using a Superscript VILO cDNA Synthesis Kit (Invitrogen). Primers used are Pannexin1 (F: AGCCAGAGAGTGGAGTTCAAAGA; R: CATTAGCAGGACGGATTCAGAA) and 18S ribosomal RNA (F: CACGGCCGGTACAGTGAAAC; R: AGAGGAGCGAGCGACCAAA). GAPDH primers (F: CAAGGCTGTGGGCAAGGTCA; R: CATCATACTTGGCAGGTTTC). *GAPDH* and *18S* were used as house-keeping genes for normalization. Real-time RT–PCR was performed using SYBR Green PCR Master Mix with 7300 Fast Real-Time PCR system (Applied Biosystems). Reaction mixtures were denatured at 95°C for 10 min, followed by 40 PCR cycles. Each cycle consisted of the following three steps: 94°C for 15 s, 57°C for 15 s, and 72°C for 1 min. Each sample was normalized against internal controls (*18S* ribosomal or *GAPDH* RNAs); the relative values for target abundance was extrapolated from standard curves generated from the reference standard.

### Immunohistochemistry

Trigeminal ganglia from WT, Panx1 KO-first, Panx1^f/f^ and conditional Panx1 KO (GFAP-Cre:Panx1^f/f^ and NFH-Cre:Panx1^f/f^) mice were removed from animals anesthetized with isoflurane and sacrificed by decapitation. Isolated trigeminal ganglia were then fixed in 4% p-formaldehyde overnight and incubated in 30% sucrose for 48 h. Tissues were then embedded in O.C.T, cryosectioned (12 μm), incubated with blocking solution containing 0.4% Triton-X, and immunostained with a Panx1 antibody and with neuronal (NeuN) or glial (glutamine synthase) markers. The primary antibodies used were: chicken anti-Panx1 (1:500; extracellular loop epitope: VQQKSSLQSES; Aves Lab #6358); mouse anti-NeuN (1:100; Millipore); goat anti-glutamine synthase (1:200; Santa Cruz). Secondary Alexa conjugated antibodies (1:2000) were: goat anti-chicken, goat anti-mouse, and donkey anti-goat. Images were acquired using an Olympus FluoView 300 confocal laser scanning microscope equipped with a 40× water-immersion lens (0.80 NA), and FITC, TRITC, and UV filter sets.

## Results

### Presence of pannexin1 mRNA in pannexin1 KO-first mice

The Panx1 transgenic mice generated by KOMP using the KO-first strategy (Testa et al., 2004) result from the insertion into intron 1 of the *Panx1* gene of a cassette containing a splice acceptor that captures the nascent RNA and a polyadenylation signal that truncates the transcript downstream of the cassette (Figure 1). Depending on the intron, this type of construct can yield either a KO or a hypomorphic allele, in case of alternative splicing or the presence of a downstream promoter.

**Figure 2:**
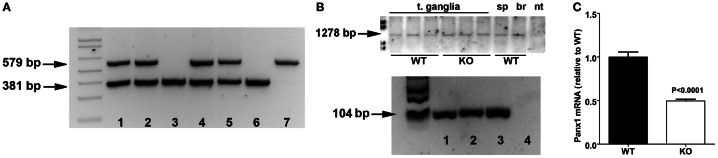
**Panx1 KO-first mice express Panx1 mRNA. (A)** Tail PCR products obtained using specific primers (primer sets F1a—R1a and F1b—R1b depicted in Figure 1) designed to detect wild-type (579 bp amplicon; lane 7), homozygous Panx1 KO-first (381 bp amplicon; lanes 3 and 6) and the heterozygous (579 and 381 bp amplicons; lanes 1, 2, 4, and 5) alleles. Data are from a litter derived from heterozygous breeding. **(B)** *Top*: RT-PCR products obtained from tissues of wild-type (WT) and Panx1 KO-first (KO) using primers (primer set F2—R2 depicted in Figure 1) designed to detect wild-type *Panx1* mRNA (1278 bp) coding region. T. ganglia, trigeminal ganglia; sp, spleen; br, brain; nt, no template. *Bottom*: PCR products obtained from RT-PCR amplicons extracted from gel on top part using primers spanning exons 4 and 5 of *Panx1* (primer set F3—R3 depicted in Figure 1). Wild-type (lane 1) and Panx1 KO-first (lane 2) trigeminal ganglia, *Panx1* cDNA plasmid (lane 3), and no template (lane 4). **(C)** Histograms of the mean ± s.e.m. values of *Panx1* mRNA expression levels detected from tail tips of wild-type (WT) and Panx1 KO-first (KO) mice by qRT-PCR using primer set F3—R3. Statistical significance obtained by unpaired *T*-test, *N* = 3 mice per group.

Heterozygous Panx1 KO-first purchased from KOMP were bred to obtain homozygous WT and Panx1 KO-first mice (Figure 2A). To evaluate whether Panx1 KO-first mice represent a complete KO or a hypomorphic phenotype with residual *Panx1* transcript, we designed RT-PCR primers (set F2—R2 in Figure 1) to detect *Panx1* mRNA coding region in tissues of WT and Panx1 KO-first adult mice. As shown in Figure 2B, Panx1 KO-first mice displayed amplicons corresponding to the entire *Panx1* mRNA region (1278 bp) similar to WT (C57Bl/6N and C57Bl/6J) mice, confirming that the KO-first strategy did not completely prevent *Panx1* transcription. In addition, extraction of bands from the RT-PCR gel for further amplification, using primer sets spanning exons 4 and 5 of *Panx1*, confirmed that the KO-first trapping cassette could be bypassed (Figure 2B, bottom), allowing for transcription of the entire *Panx1* mRNA in the Panx1 KO-first mice. Note, in Figure 2B *Panx1* mRNA expression is shown qualitatively, but not quantitatively. To quantify the extent of *Panx1* deletion (i.e., efficiency of the KO-first trapping cassette), we performed qRT-PCR using primers (set F3—R3 in Figure 1; downstream of the trapping cassette) and tail tip samples from 3 WT and 3 KO-first adult mice.

After normalization of ct values of *Panx1* to those obtained for control *18S, Panx1* mRNA expression levels recorded from Panx1 KO-first were compared to those obtained from WT samples. As indicated in Figure 2C, we measured about 50% reduction of *Panx1* mRNA in tail tips of Panx1 KO-first relative to that of WT.

Thus, our results indicate that transcription of *Panx1* mRNA is only partially ablated in the Panx1 KO-first mouse.

### Hypomorphic phenotype of pannexin1 KO-first mice

**Figure 3:**
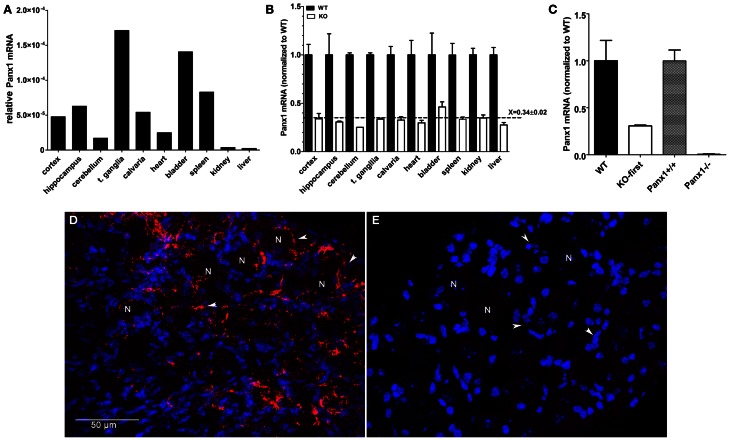
**Panx1 mRNA expression is decreased by 70% in tissues from Panx1 KO-first mice. (A)** *Panx1* mRNA expression levels (normalized to *18S* RNA) obtained by qRT-PCR from various tissues of wild-type mice using primers spanning exons 4 and 5 of *Panx1* (primer set F3—R3 depicted in Figure 1). **(B)** Bar histograms of the mean ± s.e.m. values of *Panx1* mRNA obtained by qRT-PCR of various tissues of wild-type (black bars) and Panx1 KO-first (white bars) mice. Panx1 KO-first mRNA expression levels (normalized to that of *18 S*) was calculated relative to those of wild-type. RNA was extracted from 10 different tissues of 3 mice per genotype. Dotted line represents the mean ± s.e.m. value of *Panx1* mRNA level (relative to WT) obtained from all tissues of Panx1 KO-first mice. **(C)** Bar histograms of the mean ± s.e.m. values of *Panx1* mRNA obtained by qRT-PCR of hippocampi of KOMP (WT and Panx1 KO-first) and Monyer (Panx1^+/+^ and Panx1^−/−^) mice. RNA was extracted from tissues of 3 mice per genotype and mRNA expression levels (normalized to *18 S*) was calculated relative to those of their respective wild-types. **(D,E)** Confocal images of **(D)** WT and **(E)** Panx1 KO-first trigeminal ganglia immunostained with anti-Panx1 antibody (red) and counterstained with DAPI (blue). N indicates neurons and white arrowheads indicate satellite glia cells surrounding neuronal cell bodies. Bar: 50 μm.

Pannexin1 is ubiquitously expressed, but its expression levels in different tissues have not previously been quantified. We therefore measured *Panx1* mRNA levels in various tissues of three WT mice by qRT-PCR using primers (set F3—R3 in Figure 1) spanning exons 4 and 5. Comparison of *Panx1* mRNA expression levels (normalized to *18 S*) identified highest *Panx1* levels in trigeminal ganglia of the PNS (t. ganglia; 17.12e-005), followed by bladder (14.05e-005) and spleen (10.94e00-5) (Figure 3A). *Panx1* mRNA expression levels were 4–10 times lower in the CNS compared to t. ganglia and differed in various brain regions (cortex: 4.772e-005, hippocampus: 6.28 e-005, cerebellum: 1.705e-005). Similar results were obtained when *Panx1* mRNA were normalized to *GAPDH* (data not shown).

We next evaluated by qPCR whether down-regulation of *Panx1* transcript occurred in a homogeneous fashion across different tissues from Panx1 KO-first mice. Tissues of at least 3 mice per genotype were used; no siblings were used and not all tissues were from the same set of animals. We found that *Panx1* mRNA expression was similarly reduced by 70% in all Panx1 KO-first tissues analyzed compared to their respective tissue in WT mice. Figure 3B shows *Panx1* mRNA levels in Panx1 KO-first mice normalized to those of WT mice, which are (mean ± s.e.m.): cortex (WT: 1.0 ± 0.11; KO: 0.34 ± 0.06), hippocampus (WT: 1.0 ± 0.22; KO: 0.31 ± 0.008), cerebellum (WT: 1.0 ± 0.02; KO: 0.25), trigeminal ganglia (WT: 1.0 ± 0.02; KO: 0.34 ± 0.008), calvaria (WT: 1.0 ± 0.09; KO 0.33 ± 0.034), heart (WT: 1.0 ± 0.15; KO 0.3 ± 0.18), bladder (WT: 1.0 ± 0.22; KO: 0.46 ± 0.05), spleen (WT: 1.0 ± 0.12; KO: 0.34 ± 0.22), kidney (WT: 1.0 ± 0.07; KO: 0.35 ± 0.03), and liver (WT: 1.0 ± 0.08; KO: 0.28 ± 0.03).

These results indicate that the KO-first cassette leads to a similar reduction of *Panx1* mRNA in all tissues tested, and that, for any given tissue, the variability between animals was low, as evaluated by the s.e.m. values.

Using this same set of primers and qPCR conditions, we evaluated *Panx1* levels in another transgenic mouse line, the Panx1^−/−^ (Bargiotas et al., 2011). Compared to Panx1 KO-first, *Panx1* mRNA levels measured from hippocampi of Panx1^−/−^ mice was markedly reduced (Panx1^+/+^: 0.99 ± 0.12; Panx1^−/−^: 0.01 ± 0.003; Figure 3C), further confirming the hypomorphic phenotype of the Panx1 KO-first.

Immunohistochemistry performed on trigeminal ganglia isolated from WT and Panx1 KO-first indicated that the remaining 30% Panx1 mRNA in the KO-first tissue did not result in detectable Panx1 protein (Figures 3D,E).

### Conditional, cell-specific Panx1 KO mice: GFAP-Cre:Panx1^f/f^ and NFH-Cre:Panx1^f/f^

**Figure 4:**
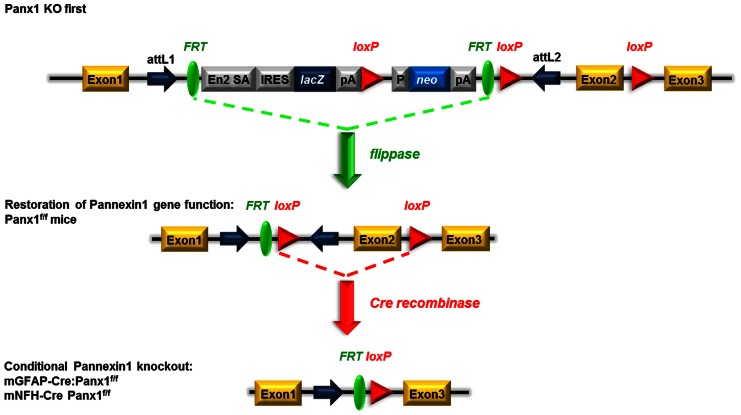
**Schematic view of the generation of conditional GFAP-Cre and NFH-Cre Panx1 ^tm1a(KOMP)Wtsi^ mice.** Mice homozygous for the “knockout-first” allele are expected to have a null phenotype due to the splicing of exon 1 to a *lacZ* trapping element and disruption of *Panx1* mRNA expression. The insertion cassette is flanked by FRT recombination sites that allow flippase recombinase to remove the gene-trapping cassette, hereby converting the “knockout-first” allele to a conditional allele (loxP sites flanking exon 2) and restoring *Panx1* gene expression/activity. Upon removal of the floxed exon 2 with Cre recombinases, transcription of this *Panx1* allele generates a frameshift mutation and premature stop codon, which triggers nonsense mediated decay of the transcript. Adapted from www.KOMP.org.

One of the advantages of the KO-first strategy is the possibility of generating conditional deletion of the gene of interest. By crossing Panx1 KO-first with flippase deleter mice, the KO-first cassette flanked by FRT sites is removed, and *Panx1* gene function is restored, leaving 2 loxP sites flanking exon 2 of *Panx1*. Crossing these floxed Panx1 mice with mice expressing Cre recombinase under cell-specific promoters allows for the generation of conditional KO mice (Figure 4).

**Figure 5:**
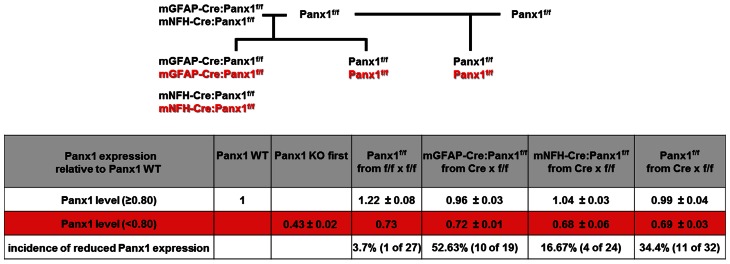
**Ectopic activity of Cre recombinase. (Top)** Breeding scheme of Panx1^f/f^ mice and conditional Panx1 knockout mice (mGFAP-Cre:Panx1^f/f^ and NFH-Cre-Panx1^f/f^) used to evaluate *Panx1* mRNA expression. **(Bottom)** Table showing *Panx1* expression determined by tail qPCR of conditional Panx1 knockout and in Panx1^f/f^ mice, which were generated as shown in top part. Animals were considered to have ectopic Cre activity if they showed less than 80% of *Panx1* mRNA expression compared to that found in WT mice.

To evaluate the extent to which *Panx1* mRNA expression was restored after removal of the KO-first cassette, we performed qRT-PCR on tail tip samples of Panx1^f/f^ and compared that to WT samples. As shown in Figure 5, we found that in 26 Panx1^f/f^ mice *Panx1* mRNA was 1.22 ± 0.08 fold that of WT mice.

We then generated conditional astrocyte- and neuron-specific Panx1 KO mice by crossing Panx1^f/f^ with either mGFAP-Cre or mNFH-Cre mice, respectively. Cre-recombinase displays high incidence of ectopic activity, leading to non-cell-type specific target deletion (Schmidt-Supprian and Rajewsky, 2007). We therefore developed a strategy to detect ectopic Cre activity in the tail of GFAP-Cre:Panx1^f/f^ and NFH-Cre:Panx1^f/f^ mice using qRT-PCR to determine *Panx1* mRNA expression levels. We considered that if mice showed tail levels of *Panx1* mRNA expression level less than 80% that of Panx1^+/+^ mice, they were likely to exhibit ectopic Cre expression, since reduced *Panx1* mRNA expression in the tail would most likely be due to off target deletion of *Panx1*. Based on this consideration, we found that 16.7% (4/24) NFH-Cre:Panx1^f/f^ and 52.6% (10/19) GFAP-Cre:Panx1^f/f^ offspring from Panx1^f/f^ females and Cre:Panx1^f/f^ males had less than 80% *Panx1* mRNA compared to WT controls (Figure 5). We found that about 34% Panx1^f/f^ littermates (offspring of Panx1^f/f^ and mNFH-Cre:Panx1^f/f^ or mGFAP-Cre:Panx1^f/f^) had lower *Panx1* mRNA expression in tail tip samples (0.69 ± 0.03 fold; *N* = 11 mice) compared to WT mice. No significant differences in *18S* RNA levels were detected among these samples. This reduced expression of *Panx1* mRNA in Panx1^f/f^ (littermates of conditional Panx1 KO mice) compared to those detected from offspring of Panx1^f/f^ and Panx1^f/f^ mice, most likely relates to the transient expression of Cre in the germlines of Panx1^f/f^ derived from GFAP-Cre or mNFH-Cre crosses.

**Figure 6:**
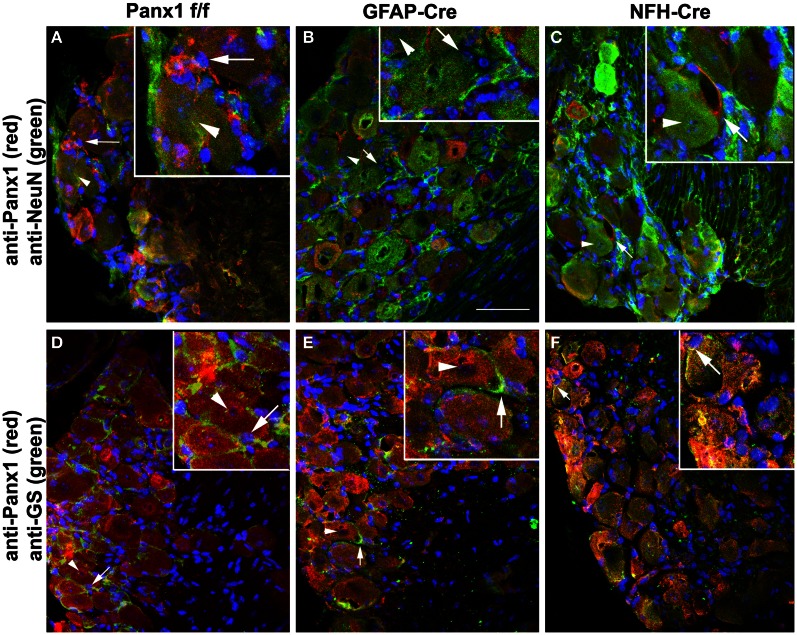
**Conditional deletion of Panx1.** Confocal images of trigeminal ganglia showing expression of Panx1 in satellite glial cells and neurons in Panx1^f/f^ **(A,D)**, GFAP-Cre:Panx1^f/f^ **(B,E)** and NFH-Cre:Panx1^f/f^ **(C,F)** mice. The cell-specific markers NeuN and glutamine synthase (GS) were used to identify neurons (arrowheads) and glial cells (arrows), respectively. Panx1 immunostaining is evident in both neurons and glial cells of Panx1^f/f^ mouse ganglion **(A,D)**. In GFAP-Cre:Panx1^f/f^ mice Panx1 immunoreactivity (red) is observed in neurons and not in glial cells **(B,E)**. Conversely, in NFH-Cre:Panx1^f/f^ mice Panx1 immunoreactivity (red) is observed in glial cells and not in neurons **(C,F)**. Note in panel **(F)** the faint immunoreactivity for Panx1 that colocalizes with neurons but actually corresponds to Panx1 expression in glial cells enwrapping the neuronal cell bodies. Images were obtained with a Olympus Fluoview 300 confocal laser scanning microscope equipped with 40× water-immersion lens (0.80 NA), U.V., and laser lines and appropriate filter sets. Bar: 50 μm; magnification in insets 2× that in main panels.

Mice that did not show ectopic Cre activity were then evaluated for Panx1 deletion in glia and neuronal cells using trigeminal ganglia processed for immunohistochemistry. As shown in Figure 6, Panx1 expression in satellite glial cells (Figures 6B,E) and in neurons (Figures 6C,F) was significantly reduced in the trigeminal ganglia of GFAP-Cre and NFH-Cre:Panx1^f/f^ mice, respectively, compared to that found in Panx1^f/f^ ganglia (Figures 6A,D).

## Discussion

Here we characterized *Panx1* expression in WT and transgenic Panx1 mice developed by KOMP. Although Panx1 is ubiquitously expressed in WT mice, we detected different levels of *Panx1* mRNA in distinct tissues, with highest *Panx1* levels in trigeminal ganglia, bladder and spleen. Transgenic Panx1 mice from KOMP were generated using a KO-first approach, which allows generation of global KO or conditional KO mice. We found that Panx1 KO-first mice represent a hypomorphic phenotype, and not a complete KO. Moreover, *Panx1* mRNA was similarly reduced by 70% in all tissues derived from Panx1 KO-first mice compared to the level of each respective WT tissue. Mice with conditional deletion of *Panx1* in astrocytes and neurons, which we generated from Panx1 KO-first mice using FLP deleter and cell-type specific Cre mice, are shown to exhibit ectopic Cre expression with non-specific Panx1 downregulation in the tail of 16.7% NFH-Cre:Panx1^f/f^ and 52.6% GFAP-Cre:Panx1^f/f^. This indicates the importance of careful monitoring of transgenic Panx1 KO mice for colony stability. To this end, we provide a useful molecular biological approach using tail biopsies.

In contrast to standard KO designs using targeted deletion, the KO-first approach leaves the *Panx1* gene intact and *Panx1* mRNA truncation depends on splicing *Panx1* exon1 to a trapping element consisting of splice acceptor and polyadenylation site contained in the targeting cassette (International Mouse Knockout Consortium et al., 2007). However, the function of this RNA processing module can be bypassed through alternative splicing due to inefficiency in the splice acceptor-polyA module or the presence of additional, downstream regulatory elements. This inefficiency is likely the cause of the hypomorphic phenotype that we report in Panx1 KO-first mice which featured a residual 30% *Panx1* mRNA in several tissues. Such hypomorphic phenotype has been previously described for other targeted genes (Meyers et al., 1998; Nagy et al., 1998).

The remaining Panx1 transcripts present in the KO-first tissues is not sufficient to revert the KO to a WT phenotype. Indeed, immunohistochemistry of trigeminal ganglia, a tissue that displays high levels of Panx1 mRNA, did not reveal the presence of Panx1 protein in the KO-first mice (Figure 3). Similarly, previous studies reported the absence of Panx1 protein in distinct tissues and cells (hippocampus, kidney, astrocytes, microglia, erythrocytes, airway epithelia cells) derived from these Panx1 KO-first mice (Seminario-Vidal et al., 2011; Qiu et al., 2011; Santiago et al., 2011; Hanner et al., 2012; Rigato et al., 2012; Suadicani et al., 2012). However, concerns regarding antibody specificity were raised in a study in which another Panx1 KO mouse line was investigated (Bargiotas et al., 2011). In that study, only one out of four Panx1 antibodies tested indicated absence of a ~47 kDa band on western blots of Panx1 KO brain tissues (Bargiotas et al., 2011). In contrast to that report, a reduction or lack of Panx1 bands on western blots of tissues from two mouse lines (KOMP and Genentech) was recently reported using five different antibodies (Cone et al., 2013). Importantly, Cone et al. (2013) provided clear evidence that differences in patterns and intensities of bands are not restricted to the antibody sources, but is also found between Panx1 KO mouse lines and between tissues of the same transgenic mouse.

At present it is premature to conclude whether or not Panx1 protein is still present in the KO-first mice or even in other Panx1 KO mouse lines. Still, even if low levels of Panx1 protein would be present, the KOMP mice are functional Panx1 KOs. Evidence of such are our recent report showing no significant differences in animal behavior, tissue and cell physiology, and even protein expression between the Panx1 KO-first and the Panx1^−/−^ mice (Santiago et al., 2011). Taking into consideration the similarities between these two mouse lines together with our present findings showing that Panx1 KO-first express significantly higher levels of Panx1 transcripts than Panx1^−/−^ mice (Figure 2), 70% knock down of Panx1 transcript appears sufficient to produce a functional KO.

As global deletions of single genes might lead to premature lethality, conditional gene disruption offers the possibility to investigate the role of a protein in specific cell-types during development. The Cre/lox system is a simple two component genetic tool, whereby under the control of a defined promoter Cre recombinase can be restricted to a specific tissue or cell-type (Nagy, 2000). However, recent reports show that Cre expression is not often strictly confined to the desired cells, often leading to spontaneously ectopic Cre activity (Eckardt et al., 2004). Furthermore, the efficiency of Cre recombination is variable and may be lost. Also, Cre expression may result in pleiotropic effects (Lee et al., 2006; Schulz et al., 2007; Wellershaus et al., 2008), including cell toxicity (Schmidt-Supprian and Rajewsky, 2007; Requardt et al., 2009). These reports showing non-homogeneity in cell-type specific target gene disruption, may have major impact on the experimental outcome. Therefore, individual mice with non-cell-type specific recombination (ectopic Cre activity) or reduced Cre activity have to be identified and discarded and the extent of the Cre-mediated gene ablation, here *Panx1*, must be correlated with phenotypic alterations.

As previously reported for the hGFAP-Cre: Cx43^f/f^ mice (Requardt et al., 2009), we reveal a limitation of GFAP-Cre:Panx1^f/f^ and NFH-Cre:Panx1^f/f^ mice, spontaneous ectopic *Panx1* gene disruption, that requires a rigorous quality control. We have observed ectopic GFAP-Cre and NFH-Cre mediated recombination, which might be caused by spontaneous transgene rearrangements (Schulz et al., 2007), or other epigenetic mechanisms suggested to lead to evolutionary selection against Cre activity (Lee et al., 2006; Requardt et al., 2009). Individual genotyping PCR to detect Cre on the DNA level does not necessarily indicate the presence or absence of gene disruption in a specific cell-type, and analyses of the efficacy of disruption is required. We suggest tail-biopsy qRT-PCR for quality control to pre-experimentally ensure that *Panx1* expression levels in the tail are unaffected by GFAP- or NFH-Cre directed Cre activity. This simple control monitors ectopic Cre activity. The Cre recombination status in the colony must be properly monitored by routine testing of the offspring obtained from all breeding pairs, to ensure cell-specific Cre activity and *Panx1* gene disruption. Otherwise, unfortunate choice of parental animals with ectopic or non-functional Cre may increase the number of these mice in the colony during the following generations. For experimental animals, a good strategy to validate cell-specific *Panx1* gene disruption is the post-experimental confirmation of cell-specific Cre activity and *Panx1* deletion using immunohistochemical approaches in every individual mouse and to correlate the index of gene inactivation with phenotypical alterations. Indeed, our immunohistochemical studies indicated significant reduction of Panx1 expression in satellite glial cells and neurons of the trigeminal ganglia of GFAP-Cre:Panx1^f/f^ and NFH-Cre:Panx1^f/f^ mice which did not show ectopic Cre expression. The lack of complete cell-type specific deletion of Panx1 in these conditional KO mice may be related to the efficiency of GFAP and NFH recombination which could be checked using a reporter mouse line. Using such an approach, we estimated that 80% astrocytes and 70% pyramidal neurons of the hippocampus are recombined when using the mGFAP-Cre and NFH-Cre lines (data not shown). These values are in accordance with previous reports using these Cre mouse lines (Hirasawa et al., 2001; Garcia et al., 2004).

Panx1 has been shown to be a major ATP release channel, which is expressed in all cells releasing ATP (Dahl and Keane, 2012). Panx1 channels also interact with other proteins, such as the purinergic receptor P2X_7_ and possibly inflammasome components involved in the innate immune response and associated secondary cell death (Pelegrin and Surprenant, 2006; Locovei et al., 2007; Silverman et al., 2009), thus responsible for amplification of the primary lesion in CNS trauma, stroke and epilepsy (Bergfeld and Forrester, 1992; De Rivero Vaccari et al., 2009; Santiago et al., 2011). Recently, it has been shown that Panx1 is involved in the fusion of T cell membrane with the human immunodeficiency virus (Seror et al., 2011). In all these processes Panx1-mediated ATP release is an early signal event in which Panx1 acts as a signal amplifier and is therefore an obvious target for the development of innovative therapeutic approaches. Advantages of Panx1 as a drug target are that it is accessible to drugs and Panx1 inhibitors are already available and FDA approved, such as the anti-malaria drug mefloquine and the gouty arthritis drug probenecid. However, more research is required to identify whether Panx1 has additional roles under physiological and pathological conditions in order to avoid undesirable side effects when targeting it.

We conclude that mice with *Panx1* modulation, specifically the multi-purpose Panx1 KO-first mice, represent a suitable model to investigate these questions. However, in this model and in many others using this strategy, the expression levels of the genes of interest must be carefully monitored to allow for a correct interpretation of the experimental findings.

### Conflict of interest statement

The authors declare that the research was conducted in the absence of any commercial or financial relationships that could be construed as a potential conflict of interest.

## Abbreviations

Panx1: pannexin1
CNS: central nervous system
PNS: peripheral nervous system
RT-PCR: reverse transcription-polymerase chain reaction
qRT-PCR: quantitative real time RT-PCR
GFAP: glia fibrillary acidic protein
NFH: neurofilament H
WT: wild-type
KO: knockout.
